# Identification of predictors for short-term recurrence: comprehensive analysis of 296 retroperitoneal liposarcoma cases

**DOI:** 10.1186/s12957-024-03328-2

**Published:** 2024-02-06

**Authors:** Zhiyuan Yu, Rui Li, Zhen Yuan, Jiahu Ye, Ping He, Peiyu Li, Yan Sun, Xudong Zhao

**Affiliations:** 1 Medical School of Chinese PLA, Beijing, China; 2 Department of General Surgery, The First Medical Center, Chinese PLA General Hospital, Beijing, China; 3 School of Medicine, Nankai University, Tianjin, China; 4 Outpatient Department of Hongshankou, Jingbei Medical District, Chinese PLA General Hospital, Beijing, China; 5 Department of General Surgery, Second Affiliated Hospital of Harbin Medical University, Heilongjiang Province, Harbin, China

**Keywords:** Retroperitoneal liposarcoma, Primary retroperitoneal liposarcoma, Short-term recurrence, Risk factors

## Abstract

**Background:**

The short-term (≤ 1 year) recurrence (STR) is the primary determinant impacting both the life quality and survival duration in patients who have undergone surgical resection for retroperitoneal liposarcoma (RPLS), a condition with intricate and ambiguous pathogenesis. The purpose of this study was to analyze the risk factors associated with STR in cases of RPLS and primary retroperitoneal liposarcoma (PRPLS).

**Methods:**

For this retrospective observational study, a total of 296 RPLS cases were selected as research subjects, who experienced tumor recurrence during the follow-up period. The Local recurrence-free survival (LRFS) rates were estimated using the Kaplan–Meier method and subsequently compared between groups utilizing the log-rank test. The subsequent analyses involved univariate and multivariate logistic regression to identify predictors of STR in RPLS cases. Additionally, a logistic regression model was constructed for PRPLS.

**Results:**

The 1-, 3-, and 5-year LRFS rates of the 296 RPLS cases were 51.7%, 16.9%, and 7.1%, respectively. In the univariate analysis, several factors were found to be associated with STR, including preoperative neutrophil/lymphocyte ratio (NLR), smoking history, surgical frequency, combined organ excision, operative time, intraoperative bleeding, transfer to the intensive care unit (ICU), multiple primary tumors, tumor shape and capsule characteristics, histological subtype, and presence of tumor necrosis. The elevated preoperative NLR, surgical frequency of ≥ 3 times, transfer to the ICU, presence of multiple primary tumors, and tumor necrosis were identified as independent risk factors for STR in surgically resected RPLS. Conversely, diabetes, intact tumor capsule, and well-differentiated histological subtype appeared to be independent protective factors. Analysis for PRPLS revealed that tumor capsule and tumor necrosis were independent predictors of STR.

**Conclusions:**

The elevated preoperative NLR, surgical frequency of ≥ 3 times, transfer to the ICU, presence of multiple primary tumors, tumor necrosis, and tumor capsule were expected to serve as predictive factors of STR for surgical resected RPLS and PRPLS.

## Introduction

Retroperitoneal malignancy refers to a group of tumors that originate from the retroperitoneal space, encompassing various tissue types and presenting with concealed clinical symptoms. Despite its rarity, accounting for less than 0.1% of all malignant tumors, retroperitoneal liposarcoma (RPLS) stands as the most prevalent soft tissue sarcoma in this anatomical region [1–3]. The deep anatomical location and rapid progression of RPLS tumors pose challenges in terms of the complexity and extent of radical surgery. Despite undergoing radical surgery, cases of primary retroperitoneal liposarcoma (PRPLS) still exhibit a high 5-year local recurrence rate ranging from 20 to 75%. The recurrence of tumors and the need for reoperation can significantly impact patients' quality of life, increase hospitalization frequency, and even result in mortality [3–5].

Existing literature evidence suggests that tumor recurrence may be attributed to the following mechanisms [6–8]: Firstly, the dense adhesion and large volume of RPLS tumors result in a loss of anatomical space, thereby increasing the surgical difficulty and the likelihood of residual tumor tissue or capsule. Secondly, tumor invasion into internal organs, blood vessels, or nerves poses significant challenges for complete resection of RPLS. Lastly, due to its resemblance to normal adipose tissue, lobulated RPLS can be mistaken as multiple tumors and resected in fragments, potentially leading to residual tumor tissue.

Although numerous studies have reported on the risk factors associated with recurrence of RPLS, the findings have been inconsistent [9–14]. Xue's study revealed that the French Federation of Cancer Centers Sarcoma Group (FNCLCC) grade and completeness of resection could serve as independent predictors for local recurrence-free survival (LRFS) [9]. Furthermore, Sun et al. discovered that recurrence, age, tumor necrosis, and tumor site were valuable prognostic markers for RPLS [12], while Yan's study demonstrated that tumor grade was an independent prognostic factor for progression-free survival (PFS) [11]. In published studies, however, there was no distinction made between short- and long-term recurrence, and the inclusion of participants with small sample sizes was noted [10]. This analysis aims to investigate the demographic, surgical, and pathological factors that predict short-term (≤ 1 year) recurrence (STR) for both RPLS and PRPLS using case data obtained from a high-volume hospital.

## Materials and methods

### Study participants

The RPLS cases treated at the First Medical Center of the Chinese People's Liberation Army (PLA) General Hospital from January 2008 to December 2021 were screened. Research subjects were selected based on the following criteria: (1) age ≥ 18 years; (2) surgical resection of RPLS and PRPLS cases, with liposarcoma confirmed by postoperative pathology; and (3) observation of tumor recurrence during the follow-up period. The exclusion criteria were as follows: (1) neoplasms originating from adipose tissue outside the retroperitoneum; (2) patients who underwent palliative surgery or non-operative therapy; (3) cases of in-hospital mortality; and (4) cases where participation was refused or lost to follow-up. This study was conducted in accordance with the Declaration of Helsinki and approved by the Medical Ethics Committee of the First Medical Center of the Chinese PLA General Hospital.

### Data collection and outcome evaluation

According to previous studies, the risk factors associated with neoplasm recurrence encompass demographic, surgical, and pathological characteristics. Therefore, for this study, the required case data included gender, age, preoperative neutrophil/lymphocyte ratio (NLR), smoking history, diabetes, clinical symptoms, surgical frequency, tumor resection method, combined organ excision, operative time, intraoperative bleeding, application of intraperitoneal chemotherapy drug, transfer to intensive care unit (ICU), tumor diameter, multiple primary tumors, tumor shape, tumor capsule, histological subtype, and tumor necrosis.

In cases of retroperitoneal tumors with uncertain excision margins, intraoperative pathological examination was performed to ensure the efficacy of radical surgery. Palliative resection was considered in the presence of any residual tumor during the operation, and patients who underwent palliative surgery were excluded from this study. The excision of related organs, either partially or completely, was performed in cases where the tumors invaded surrounding organs. Segmented tumor resection was considered as an alternative only when complete resection while maintaining tumor integrity was not feasible. During the post-operative follow-up, patients underwent regular reviews every 3–4 months for the first two years and then every 6 months. Routine imaging techniques such as computerized tomography (CT) or abdominopelvic magnetic resonance imaging (MRI) were employed to monitor tumor recurrence. Based on the time interval between surgery and tumor recurrence, the included cases of RPLS were categorized into two groups: STR group (≤ 1 year) and non-STR group (> 1 year).

### Statistical analysis

All the statistical analyses in this study were performed using SPSS software version 26.0. Categorical data was reported as numbers (percentages), and compared using a Chi-square or Fisher’s exact test. The normality of continuous variables were checked by drawing and visually inspecting the histograms and Q-Q plots. Continuous normally distributed variables were expressed as mean with the standard deviation (SD) and compared between groups using an independent-samples T-test. Continuous not normally distributed variables were expressed as median with the interquartile range (IQR) and compared between groups using a Mann–Whitney U test. The LRFS was calculated according to the interval between surgery and tumor recurrence. Besides, we estimated the LRFS rates based on Kaplan–Meier method, and compared different groups using log-rank test. A univariate analysis was conducted to examine the correlation between covariates and dependent variables. Subsequently, the covariates with *P* < 0.1 in the univariate analysis would be included in a multivariate logistic regression model to identify the independent predictors. All tests were considered statistically significant if *P* values < 0.05.

## Results

### Patients characteristics

**Fig. 1:**
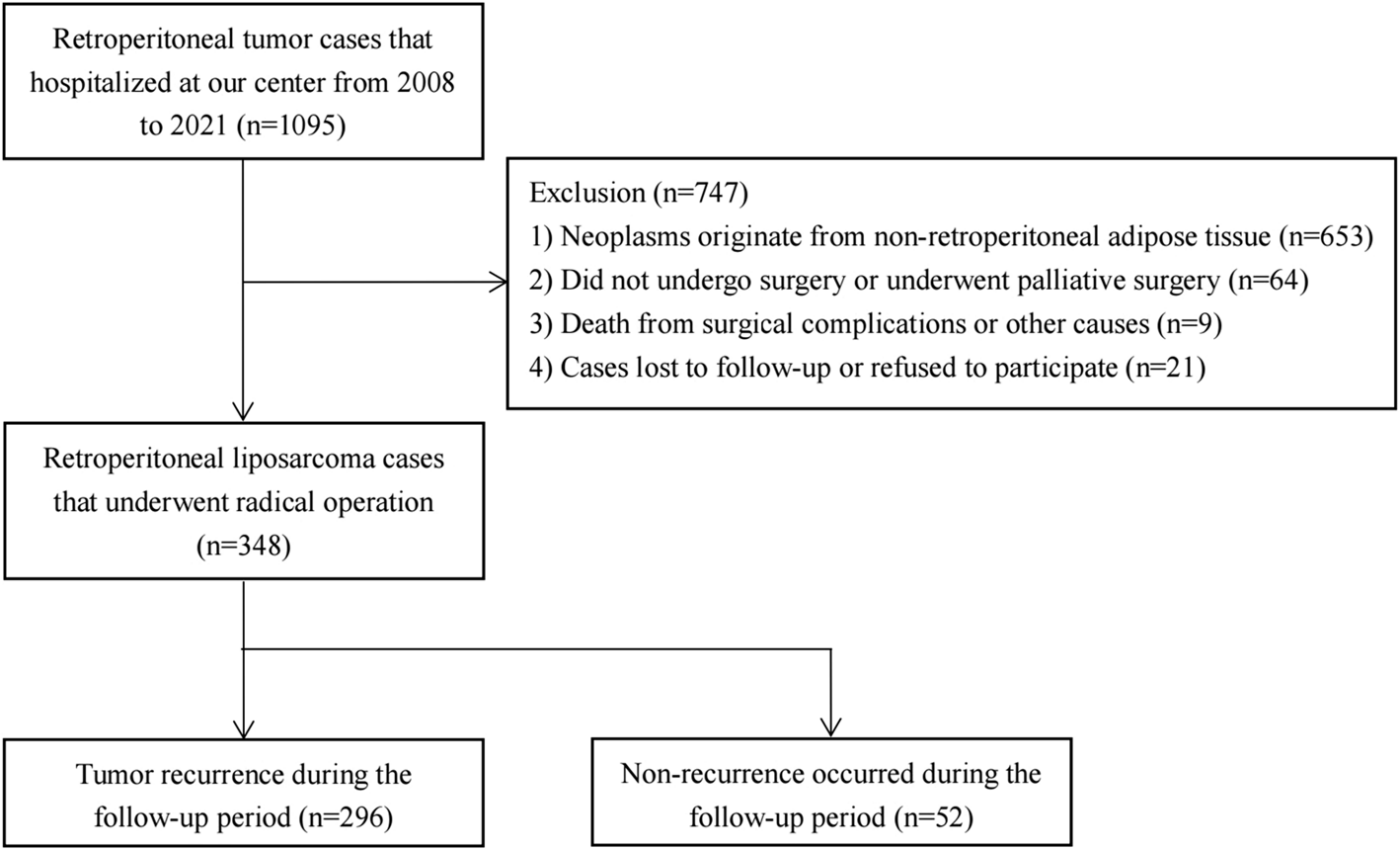
The flowchart of patient selection

After screening, a total of 1095 in-hospital cases of RPLS were identified at the study center from January 2008 to December 2021 using the electronic medical records (EMR) system. Among these patients, 653 neoplasms originated from adipose tissue outside the retroperitoneum, while 64 individuals received palliative surgery or non-operative therapy. Additionally, nine patients experienced mortality during their hospital stay, and 21 either refused to participate or were lost to follow-up. Furthermore, there were 52 cases without tumor recurrence. Ultimately, a total of 296 RPLS cases with tumor recurrence during the follow-up period were included as research subjects in this analysis (Fig. 1). The median LRFS for these 296 RPLS cases was 13.0 months [95% confidence interval (CI): 10.8–15.2], and the LRFS rates at 1, 3, and 5 years were found to be 51.7%, 16.9%, and 7.1%, respectively. Among the cohort of patients, there were initially diagnosed cases (*n* = 94) and relapse cases (*n* = 202), with median LRFS durations of 23.0 months (95% CI:17.3–28.7) and11 0.0 months(95% CI:9 0.9–12 0.1), respectively.

### Logistic regression analyses for 296 RPLS cases

The 296 cases of RPLS were divided into two groups based on the duration of LRFS: STR group (≤ 1 year) and non-STR group (> 1 year), which included 143 and 153 cases, respectively. Univariate analysis revealed that preoperative NLR (*p* = 0.004), smoking history (*p* = 0.026), surgical frequency (*p* < 0.001), combined organ excision (*p* = 0.049), operative time (*p* < 0.001), intraoperative blood loss (*p* < 0.001), transfer to ICU (*p* = 0.001), multiple primary tumors (*p* < 0.001), tumor shape (*p* = 0.024), tumor capsule status (*p* < 0.001), histological subtype (*p* < 0.00 l), and presence of tumor necrosis (*p* < 0.00 l) were significant predictive factors for achieving STR. The association between STR and gender, age, BMI, diabetes, clinical symptoms, resection method, application of intraperitoneal chemotherapy drug, or tumor diameter was not found to be statistically significant (*p* > 0.05) (Table 1).

**Table 1:** Characteristics of included RPLS cases in STR group and non-STR group

Variables	Total (*n* = 296)		STR group (*n* = 143)	non-STR group (*n* = 153)	*p* value
Demographic characteristics					
Gender					0.539
Male	171 (57.8)		80 (55.9)	91 (59.5)	
Female	125 (41.2)		63 (44.1)	62 (40.5)	
Age (years)	53 (46, 60)		54 (47, 60)	53 (45, 61)	0.321
Preoperative NLR	3.08 (1.89, 3.58)		3.57 (2.20, 3.58)	2.40 (1.69, 3.58)	0.004
BMI (kg/m^2^)	23.55 (21.80, 24.80)		23.55 (22.06, 24.61)	23.55 (21.53, 25.51)	0.472
Smoking history					0.026
Yes	50 (16.9)		17 (11.9)	33 (21.6)	
No	246 (83.1)		126 (88.1)	120 (78.4)	
Diabetes					0.085
Yes	15 (5.1)		4 (2.8)	11 (7.2)	
No	281 (94.9)		139 (97.2)	142 (92.8)	
Clinical symptoms					0.653
Yes	172 (58.1)		85 (59.4)	87 (56.9)	
No	124 (41.9)		58 (40.6)	66 (43.1)	
Surgical characteristics					
Surgical frequency					< 0.001
1st time	94 (31.8)		28 (19.6)	66 (43.1)	
2nd time	80 (27.0)		38 (26.6)	42 (27.5)	
≥ 3rd time	122 (41.2)		77 (53.8)	45 (29.4)	
Resection method					0.567
Piecemeal	36 (12.2)		19 (13.3)	17 (11.1)	
Complete	260 (87.8)		124 (86.7)	136 (88.9)	
Combined organ excision					0.049
Yes	201 (67.9)		105 (73.4)	96 (62.7)	
No	95 (32.1)		38 (26.6)	57 (37.3)	
Operative time (min)	239 (174, 301)		260 (196, 335)	215 (160, 280)	< 0.001
Intraoperative blood loss (ml)	800 (400, 1575)		1000 (500, 2000)	700 (300, 1200)	< 0.001
Intraperitoneal chemotherapy drug application					0.414
Yes		121 (40.9)	55 (38.5)	66 (43.1)	
No		175 (59.1)	88 (61.5)	87 (56.9)	
Transfer to ICU					0.001
Yes		74 (25.0)	48 (33.6)	26 (17.0)	
No		222 (75.0)	95 (66.4)	127 (83.0)	
Pathological characteristics					
Tumor diameter (cm)		23.0 (16.1, 30.0)	23.0 (16.5, 30.0)	22.0 (16.0, 30.0)	0.700
Multiple primary tumors					< 0.001
Yes		151 (51.0)	88 (61.5)	63 (41.2)	
No		145 (49.0)	55 (38.5)	90 (58.8)	
Tumor shape					0.024
Irregular		115 (38.9)	65 (45.5)	50 (32.7)	
Regular		181 (61.1)	78 (54.5)	103 (67.3)	
Tumor capsule					< 0.001
Intact		176 (59.5)	70 (49.0)	106 (69.3)	
Broken		120 (40.5)	73 (51.0)	47 (30.7)	
Histological subtype					< 0.001
Well-differentiated		69 (23.3)	16 (11.2)	53 (34.6)	
De-differentiated		64 (21.6)	47 (32.9)	17 (9.2)	
Other subtypes		163 (55.1)	80 (55.9)	83 (56.2)	
Tumor necrosis					< 0.001
Yes		70 (23.6)	49 (34.3)	21 (13.7)	
No		226 (76.4)	94 (65.7)	132 (86.3)	

Data presented in median (interquartile range) for continuous variables and frequency (percent) for categorical variables, *RPLS* Retroperitoneal liposarcoma, *STR* Short-term recurrence, *BMI* Body mass index, *NLR* Neutrophil/lymphocyte ratio, *ICU* Intensive care unit

**Fig.2:**
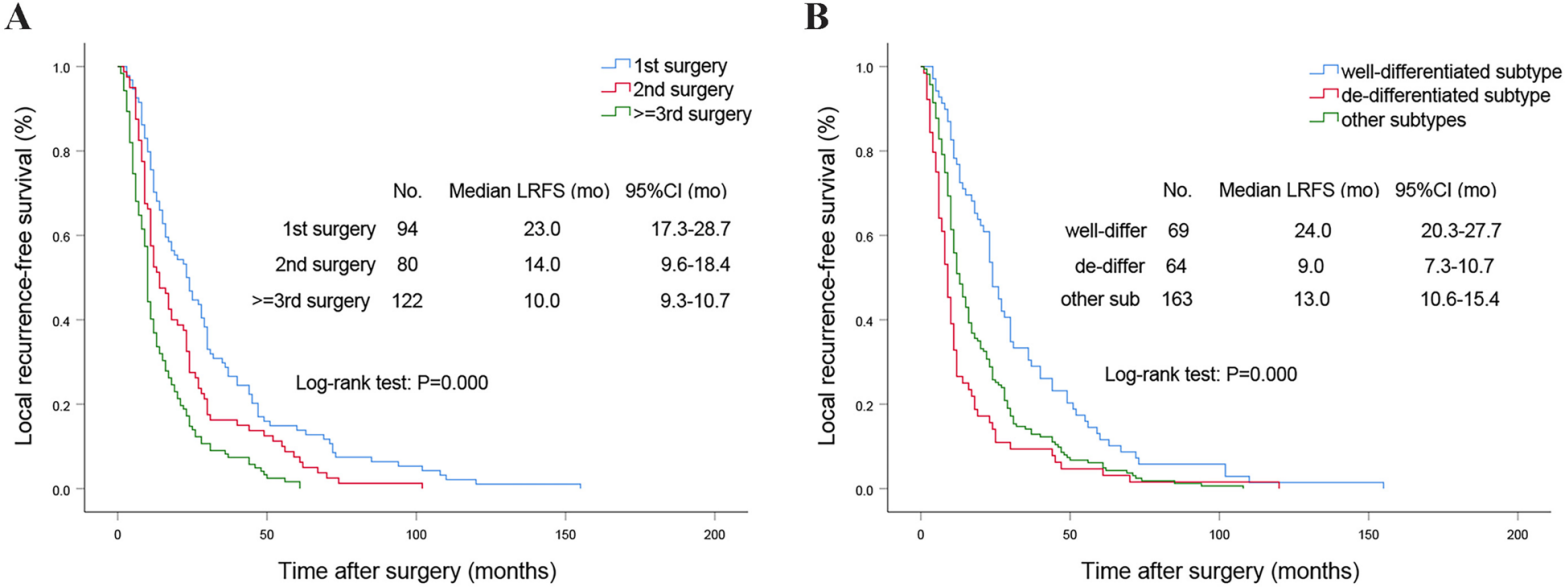
Kaplan–Meier curves of local recurrence-free survival (LRFS) for (**A**) surgical frequency, and (**B**) histological subtype

The subsequent step involved constructing a multivariate binary logistic regression model, incorporating the following variables: preoperative NLR, smoking history, diabetes, surgical frequency, combined organ excision, operative time, intraoperative blood loss, transfer to ICU, multiple primary tumors, tumor shape, tumor capsule status, histological subtype, and presence of tumor necrosis. Analysis results demonstrated that an elevated preoperative NLR [odds ratio (OR) = 1.129, *p* = 0.007], surgical frequency ≥ 3 times (OR = 2.642, *p* = 0.018), transfer to ICU (OR = 2.390, *p* = 0.013), presence of multiple primary tumors (OR = 2.052, *p* = 0.038) and existence of tumor necrosis (OR = 2.131, *p* = 0.048) were identified as independent risk factors for STR, while diabetes (OR = 0.143, *p* = 0.013), intact tumor capsule (OR = 0.499, *p* = 0.030), and well-differentiated histological subtype (*p* < 0.001) seemed to be independent protective factors (Table 2). The histological subtype and surgical frequency emerged as significant independent factors influencing tumor recurrence. Consequently, we generated Kaplan–Meier curves for LRFS based on surgical frequency (Fig. 2A) and histological subtype (Fig. 2B). Furthermore, the log-rank test results demonstrated a strong correlation between surgical frequency (*p* < 0.001) and histological subtype (*p* < 0.001) with LRFS.

**Table 2:** Multivariate analysis of predictive factors for STR among 296 RPLS cases

Variables	Coefficients	OR (95% CI)	*p* value
Demographic characteristics			
Preoperative NLR	0.121	1.129 (1.035–1.232)	0.007
Smoking history	-0.685	0.504 (0.238–1.069)	0.074
Diabetes	-1.945	0.143 (0.031–0.659)	0.013
Surgical characteristics			
Surgical frequency			0.038
2nd time vs. 1st time	0.272	1.313 (0.582–2.960)	0.511
≥ 3rd time vs. 1st time	0.971	2.642 (1.180–5.913)	0.018
Combined organ excision	-0.189	0.828 (0.421–1.626)	0.583
Operative time	0.001	1.001 (0.997–1.005)	0.717
Intraoperative blood loss	< 0.001	1.000 (1.000–1.001)	0.235
Transfer to ICU	0.871	2.390 (1.200–4.760)	0.013
Pathological characteristics			
Multiple primary tumors	0.719	2.052 (1.039–4.054)	0.038
Irregular tumor shape	0.410	1.507 (0.815–2.788)	0.191
Intact tumor capsule	-0.694	0.499(0.267–0.936)	0.030
Histological subtype			< 0.001
De-differentiated vs. well-differentiated	2.239	9.381(3.585–24.548)	< 0.001
Other subtypes vs. well-differentiated	1.040	2.831(1.326–6.043)	0.007
Tumor necrosis	0.757	2.131 (1.007–4.511)	0.048
Constant values	-2.660	0.070	< 0.001

*STR* Short-term recurrence, *RPLS* Retroperitoneal liposarcoma, *NLR* Neutrophil/lymphocyte ratio, *ICU* Intensive care unit, *OR* Odds ratio, *CI* Credible interval

### Logistic regression analyses for 94 PRPLS cases

In this study, 94 patients with RPLS were identified as initial cases, while 202 were classified as relapse cases. Subsequently, a comprehensive analysis of STR was conducted among the PRPLS cases. The results of univariate analysis demonstrated significant associations between STR for initial cases and several factors, including intraoperative blood loss (OR = 1.001, *p* = 0.006), transfer to the ICU (OR = 4.098, *p* = 0.008), presence of an intact tumor capsule (OR = 0.231, *p* = 0.004), de-differentiated histological subtype (OR = 5.914, *p* = 0.012), and the presence of tumor necrosis (OR = 6.000, *p* < 0.001).

A multivariate logistic regression model was constructed using the following variables: preoperative NLR, clinical symptoms, type of resection, duration of surgery, amount of intraoperative blood loss, transfer to ICU, presence of multiple primary tumors, intact tumor capsule status, histological subtype, and presence of tumor necrosis. In the multivariate analysis, intact tumor capsule (OR = 0.230, *p* = 0.037) and tumor necrosis (OR = 4.375, *p* = 0.026) were identified as the independent predictors for STR (Table 3).

**Table 3:** Univariate and multivariate analyses for STR among 94 PRPLS cases

Variables	Univariate analysis			Multivariate analysis		
	OR	95% CI	*p* value	OR	95% CI	*p* value
Demographic characteristics						
Gender	0.923	0.377–2.259	0.861			
Age	1.027	0.987–1.070	0.190			
Preoperative NLR	1.029	0.934–1.134	0.567	1.055	0.932–1.194	0.399
BMI (kg/m^2^)	0.949	0.825–1.092	0.463			
Smoking history	0.886	0.283–2.776	0.836			
Diabetes	0.769	0.146–4.067	0.757			
Clinical symptoms	2.702	0.968–7.539	0.058	2.794	0.771–10.124	0.118
Surgical characteristics						
Resection method	0.297	0.073–1.202	0.089	0.746	0.102–5.470	0.773
Combined organ excision	1.842	0.709–4.783	0.210			
Operative time	1.005	0.999–1.011	0.078	1.000	0.991–1.009	0.970
Intraoperative blood loss	1.001	1.000–1.002	0.006	1.000	0.999–1.001	0.481
Intraperitoneal chemotherapy drug application	0.978	0.403–2.374	0.962			
Transfer to ICU	4.098	1.457–11.526	0.008	3.533	0.777–16.064	0.102
Pathological characteristics						
Tumor diameter	0.989	0.945–1.034	0.612			
Multiple primary tumors	1.359	0.476–3.878	0.566	0.677	0.153–3.001	0.608
Irregular tumor shape	0.829	0.324–2.119	0.695			
Intact tumor capsule	0.231	0.086–0.618	0.004	0.230	0.058–0.915	0.037
Histological subtype			0.035			0.332
De-differentiated vs. well-differentiated	5.914	1.484–23.564	0.012	2.690	0.454–15.932	
Other subtypes vs. well-differentiated	1.789	0.568–5.635	0.320	0.894	0.204–3.915	
Tumor necrosis	6.000	2.262–15.914	< 0.001	4.375	1.198–15.975	0.026

*STR* Short-term recurrence, *PRPLS* Primary retroperitoneal liposarcoma, *NLR* Neutrophil /lymphocyte ratio, *BMI* Body mass index, *ICU* Intensive care unit, *OR* Odds ratio, *CI* Credible interval

## Discussion

Currently, radical surgery remains the primary treatment for RPLS, while the therapeutic efficacy of radiotherapy, chemotherapy, and targeted therapy is suboptimal [2, 4, 5, 15]. Previous studies have indicated that even well-differentiated RPLS exhibited a disease-specific mortality rate ranging from 30 to 50%, whereas de-differentiated RPLS presented an alarmingly high disease-specific mortality rate of up to 30%-75% [16]. The identification of risk factors for postoperative recurrence, particularly STR, remains complex and unclear in both RPLS and PRPLS cases, making it the leading cause of mortality. Hence, this study was conducted to ascertain predictors for STR.

The findings of this analysis indicated that a surgical frequency of ≥ 3 times and a de-differentiated histological subtype were likely to be significant risk factors for STR. In general, multiple surgeries necessitate careful consideration of the timing and extent of the operation, as well as the expertise and technology of the surgeons involved, along with effective collaboration within the surgical team. The increased complexity, difficulty, and associated risks of surgery could result in higher intraoperative blood loss, prolonged operative duration, and an elevated incidence of combined organ excision and transfer to the ICU, thereby increasing the likelihood of residual tumor tissue and implantation of tumor cells [6, 17, 18]. Compared to previous reports, the rate of combined organ resection reported in this study appeared to be relatively lower. On one hand, variations in the incidence, characteristics, therapeutic approach, and timing of surgery for retroperitoneal tumors across different countries and regions may contribute to this observation. On the other hand, it is important to note that the data analyzed in this study were derived from a single high-volume hospital in China, which could introduce information bias and potentially impact the overall quality of evidence.

In the past, our center adopted a more aggressive treatment strategy. For confirmed retroperitoneal tumors, radical resection was performed regardless of tumor size or invasion of surrounding organs. The literature extensively reported the importance of combined organ resection for retroperitoneal liposarcoma in reducing local recurrence rates, which was also recommended by expert consensus both domestically and internationally. In clinical practice, we have observed that combined organ resection for retroperitoneal tumors invading surrounding organs may lead to a prolonged LRFS time, however, it did not significantly reduce the risk of local recurrence. Moreover, expanding the excision scope in pursuit of radical efficacy could potentially elevate surgical risks and compromise organ functionality. Therefore, with the primary objective of ensuring surgical safety, it is imperative to perform en bloc excision of the tumor and its surrounding tissue as extensively as possible in order to minimize the risk of residual tumor presence and subsequent recurrence. In cases where piecemeal resection is unavoidable for RPLS patients, we meticulously evaluated each surgical margin intraoperatively to prevent any remnants of the tumor. Consequently, some cases of piecemeal resection were included in this study as appropriate subjects, and the local recurrence rate of these cases was not higher than that of complete resection cases [19–21].

The RPLS cases included in this study were categorized into well-differentiated, de-differentiated, and other pathological subtypes, with the latter encompassing mucous, pleomorphic, and mixed liposarcoma. Well-differentiated and de-differentiated subtypes predominated among retroperitoneal liposarcomas. However, a significant number of RPLS cases exhibited multiple pathological subtypes. In order to eliminate the potential confounding effects of different pathological subtypes, cases of RPLS that exhibited two or more subtypes were classified as mixed liposarcoma. Out of the 163 RPLS cases with other subtypes, 108 were identified as mixed liposarcoma, predominantly consisting of well-differentiated or de-differentiated components in conjunction with other subtypes. The prognosis of RPLS cases varies significantly among different histological subtypes, as reported in the majority of published literature. Notably, well-differentiated RPLS exhibited a lower recurrence rate and significantly prolonged LRFS compared to other subtypes [8, 9, 22]. Furthermore, this study demonstrates that the well-differentiated group has a lower STR rate than the de-differentiated group. The degree of tumor differentiation, the number of mitotic figures, and the extent of tumor necrosis serve as the primary criteria for tumor pathological grading. Furthermore, a low degree of tumor differentiation, a high number of mitotic figures, and extensive tumor necrosis could contribute to a higher grade of tumor, thereby increasing the incidence rate of STR [9, 11, 12, 21, 22]. The presence of irregular tumor shape, incomplete tumor capsule, and multiple lesions often accompanies de-differentiated RPLS, rendering the identification of the boundary between the tumor and normal tissue, challenging and impeding the achievement of complete radical resection [11, 23, 24].

Tumor-related inflammation can induce the tumor itself or surrounding cells to express a variety of molecules, thereby creating a microenvironment that potentially facilitates tumor progression. Elevated NLR levels, as indicated by the expression ratio of serum lymphocytes and neutrophils, typically signify the suppression of anti-tumor immune status and activation of an inflammatory response [25–27]. Therefore, the NLR level may regulate tumor cell growth rate and invasiveness, thereby influencing the incidence of STR. Moreover, this study suggested that diabetes may exert a protective effect against STR. It is widely recognized that tumor cells predominantly rely on glycolysis for energy generation in both aerobic and anaerobic environments, a phenomenon commonly referred to as the "Warburg effect". For individuals with diabetes, the disruption of glucose metabolism and the reduction in dietary sugar intake may impede the glycolysis process of tumor cells, thereby potentially decelerating the growth rate of RPLS and diminishing the risk of STR [28]. Given the limited number of diabetes cases included in this analysis, further investigation is warranted to explore its protective effect against STR.

The presence of tumor necrosis and an intact tumor capsule were identified as significant risk factors for STR in both RPLS and PRPLS cases. An intact tumor capsule often indicates a lower degree of malignancy, facilitating the achievement of complete radical resection and thereby reducing the likelihood of residual tumor tissue. Consequently, ensuring the preservation of the integrity of the initially operated tumor is particularly crucial in preventing STR in PRPLS [14]. The occurrence of tumor necrosis is often attributed to the rapid growth and chronic ischemic injury of solid tumors, which are indicative of hypoxia and high malignancy. Extensive tumor necrosis generally signifies a poor prognosis and a high likelihood of recurrence [12, 29, 30]. Therefore, it is recommended to perform intraoperative pathological examination for confirmation of the presence and extent of tumor necrosis. The presence of tumor necrosis in RPLS cases necessitates the appropriate expansion of excision scope, meticulous surgical techniques, and thorough examination to prevent residual tumor tissue. Additionally, a shortened review interval and increased frequency of postoperative reviews are essential for detecting any potential STR.

Although this study has identified risk factors associated with STR in RPLS and PRPLS cases by utilizing the case data from one of the nation's largest medical centers, there are still certain limitations that need to be acknowledged. First, the level of evidence was constrained by administrative and technical disparities between retrospective cohort studies and single-center cases, limiting the robustness of the findings. Secondly, the inclusion of case data spanning a significant period of time may contribute to the presence of information bias. Subsequently, multi-center, large-scale, and prospective studies are needed to validate and augment the findings of this study.

## Conclusion

Elevated preoperative NLR, surgical frequency of ≥ 3 times, transfer to the ICU, presence of multiple primary tumors, and tumor necrosis were identified as independent risk factors for STR in surgically treated RPLS. Conversely, diabetes mellitus, intact tumor capsule, and well-differentiated histological subtype were identified as independent protective factors. The intact tumor capsule and presence of tumor necrosis can serve as independent predictors for STR in PRPLS cases. This study has identified demographic, surgical, and pathological factors that can predict STR in both RPLS and PRPLS cases, thereby offering potential applications in clinical practice.

## Data Availability

The datasets used and/or analyzed during the current study are available from the corresponding author on reasonable request.

## Abbreviations

STR: Short-term recurrence
RPLS: Retroperitoneal liposarcoma
PRPLS: Primary retroperitoneal liposarcoma
LRFS: Local recurrence-free survival
NLR: Neutrophil/lymphocyte ratio
ICU: Intensive care unit
PFS: Progression-free survival
PLA: People's Liberation Army
EMR: Electronic medical record
CT: Computerized tomography
MRI: Magnetic resonance imaging
IQR: Interquartile range
CI: Confidence interval
OR: Odds ratio

## References

[1] Bagaria SP, Gabriel E, Mann GN (2018). Multiply recurrent retroperitoneal liposarcoma. J Surg Oncol.

[2] Istl AC, Gronchi A (2022). Neoadjuvant Therapy for Primary Resectable Retroperitoneal Sarcomas-Looking Forward. Cancers (Basel).

[3] Muratori F, Frenos F, Bettini L, Matera D, Mondanelli N, Scorianz M (2018). Liposarcoma: Clinico-pathological analysis, prognostic factors and survival in a series of 307 patients treated at a single institution. J Orthop Sci.

[4] Salerno KE, Baldini EH (2022). Role of Radiation Therapy in Retroperitoneal Sarcoma. J Natl Compr Canc Netw.

[5] Molina G, Hull MA, Chen YL, DeLaney TF, De Amorim BK, Choy E (2016). Preoperative radiation therapy combined with radical surgical resection is associated with a lower rate of local recurrence when treating unifocal, primary retroperitoneal liposarcoma. J Surg Oncol.

[6] Lewis JJ, Leung D, Woodruff JM, Brennan MF (1998). Retroperitoneal soft-tissue sarcoma: analysis of 500 patients treated and followed at a single institution. Ann Surg.

[7] Neuhaus SJ, Barry P, Clark MA, Hayes AJ, Fisher C, Thomas JM (2005). Surgical management of primary and recurrent retroperitoneal liposarcoma. Br J Surg.

[8] Singer S, Antonescu CR, Riedel E, Brennan MF (2003). Histologic subtype and margin of resection predict pattern of recurrence and survival for retroperitoneal liposarcoma. Ann Surg.

[9] Xue G, Wang Z, Li C, Lv A, Tian X, Wu J (2021). A novel nomogram for predicting local recurrence-free survival after surgical resection for retroperitoneal liposarcoma from a Chinese tertiary cancer center. Int J Clin Oncol.

[10] Sánchez-Hidalgo JM, Rufián-Peña S, Durán-Martínez M, Durán-Martínez M, Arjona-Sánchez Á, Salcedo-Leal I (2018). Risk factors of early recurrence in retroperitoneal liposarcoma. Cir Esp (Engl Ed).

[11] Yan Y, Xia S, Teng D, Hu S, Li S, Wang Y (2020). Resection outcomes for primary and local recurrent retroperitoneal liposarcoma patients. Ann Transl Med.

[12] Sun P, Ma R, Liu G, Wang L, Chang H, Li Y (2021). Pathological prognostic factors of retroperitoneal liposarcoma: comprehensive clinicopathological analysis of 124 cases. Ann Transl Med.

[13] Wu YX, Liu JY, Liu JJ, Yan P, Tang B, Cui YH (2018). A retrospective, single-center cohort study on 65 patients with primary retroperitoneal liposarcoma. Oncol Lett.

[14] Ishii K, Yokoyama Y, Nishida Y, Koike H, Yamada S, Kodera Y (2020). Characteristics of primary and repeated recurrent retroperitoneal liposarcoma: outcomes after aggressive surgeries at a single institution. Jpn J Clin Oncol.

[15] Suarez-Kelly LP, Baldi GG, Gronchi A (2019). Pharmacotherapy for liposarcoma: current state of the art and emerging systemic treatments. Expert Opin Pharmacother.

[16] Gronchi A, Miah AB, Dei Tos AP, Abecassis N, Bajpai J, Bauer S (2021). Soft tissue and visceral sarcomas: ESMO-EURACAN-GENTURIS Clinical Practice Guidelines for diagnosis, treatment and follow-up. Ann Oncol.

[17] Honoré C, Faron M, Mir O, Haddag-Miliani L, Dumont S, Terrier P (2018). Management of locoregional recurrence after radical resection of a primary nonmetastatic retroperitoneal soft tissue sarcoma: The Gustave Roussy experience. J Surg Oncol.

[18] Sato T, Yamaguchi T, Azekura K, Ueno M, Ohyama S, Ohya M (2006). Repeated resection for intra-abdominal and retroperitoneal liposarcomas: long-term experience in a single cancer center in Japan. Int Surg.

[19] Abdelfatah E, Guzzetta AA, Nagarajan N, Wolfgang CL, Pawlik TM, Choti MA (2016). Long-term outcomes in treatment of retroperitoneal sarcomas: A 15 year single-institution evaluation of prognostic features. J Surg Oncol.

[20] Homsy P, Blomqvist C, Heiskanen I, Vikatmaa L, Tukiainen E, Numminen K (2020). Multidisciplinary Oncovascular Surgery is Safe and Effective in the Treatment of Intra-abdominal and Retroperitoneal Sarcomas: A Retrospective Single Centre Cohort Study and a Comprehensive Literature Review. Eur J Vasc Endovasc Surg.

[21] Masaki N, Onozawa M, Inoue T, Kurobe M, Kawai K, Miyazaki J (2021). Clinical features of multiply recurrent retroperitoneal liposarcoma: A single-center experience. Asian J Surg.

[22] Sbaraglia M, Bellan E, Dei Tos AP (2021). The 2020 WHO Classification of Soft Tissue Tumours: news and perspectives. Pathologica.

[23] Littau MJ, Kulshrestha S, Bunn C, Agnew S, Sweigert P, Luchette FA (2021). The importance of the margin of resection and radiotherapy in retroperitoneal liposarcoma. Am J Surg.

[24] Dehner CA, Hagemann IS, Chrisinger JSA (2021). Retroperitoneal Dedifferentiated Liposarcoma. Am J Clin Pathol.

[25] Simonaggio A, Elaidi R, Fournier L, Fabre E, Ferrari V, Borchiellini D (2020). Variation in neutrophil to lymphocyte ratio (NLR) as predictor of outcomes in metastatic renal cell carcinoma (mRCC) and non-small cell lung cancer (mNSCLC) patients treated with nivolumab. Cancer Immunol Immunother.

[26] Aloe C, Wang H, Vlahos R, Irving L, Steinfort D, Bozinovski S (2021). Emerging and multifaceted role of neutrophils in lung cancer. Transl Lung Cancer Res.

[27] Guthrie GJ, Charles KA, Roxburgh CS, Horgan PG, McMillan DC, Clarke SJ (2013). The systemic inflammation-based neutrophil-lymphocyte ratio: experience in patients with cancer. Crit Rev Oncol Hematol.

[28] Huangyang P, Li F, Lee P, Nissim I, Weljie AM, Mancuso A (2020). Fructose-1,6-Bisphosphatase 2 Inhibits Sarcoma Progression by Restraining Mitochondrial Biogenesis. Cell Metab.

[29] Zhuang A, Wu Q, Tong H, Zhang Y, Lu W (2021). Development and Validation of a Nomogram for Predicting Recurrence-Free Survival of Surgical Resected Retroperitoneal Liposarcoma. Cancer Manag Res.

[30] Xiao J, Liu J, Chen M, Liu W, He X (2021). Diagnosis and Prognosis of Retroperitoneal Liposarcoma: A Single Asian Center Cohort of 57 Cases. J Oncol.

